# The role of vitamin D in slipped capital femoral epiphysis in children and adolescents: a retrospective case-control study

**DOI:** 10.3389/fendo.2024.1497103

**Published:** 2025-01-06

**Authors:** Peng Ning, Shuting Lin, Huiyu Geng, Tianjing Liu

**Affiliations:** Department of Pediatric Orthopaedics, ShengJing Hospital of China Medical University, Shenyang, China

**Keywords:** slipped capital femoral epiphysis (SCFE), severity, hormone, adolescent, 25 hydroxyvitamin D

## Abstract

**Objective:**

To explore the correlation between vitamin D levels, related endocrine/metabolic factors, and the risk of slipped capital femoral epiphysis (SCFE) in children and adolescents, and to assess whether vitamin D levels are associated with SCFE severity.

**Methods:**

A retrospective case-control study was conducted from March 2014 to October 2023 in Shengjing hospital. Patients diagnosed with SCFE were categorized as the SCFE group. The control group consisted of healthy children matched by gender, age, weight, height, body mass index (BMI), and date of blood tests at a 1:2 ratio from the pediatric developmental clinic. The analysis included relevant laboratory tests such as 25-hydroxyvitamin D (25(OH)D), hemoglobin (Hb), serum alkaline phosphatase (ALP), calcium (Ca), phosphorus (P), and magnesium (Mg), etc. Univariate and multivariate conditional logistic regression analyses were conducted to identify factors associated with SCFE, with a particular focus on the correlation between 25(OH)D levels and the risk of SCFE. The study also explored whether these factors were correlated with SCFE severity, determined by measuring the slip angle and displacement on the anteroposterior pelvic or frog-leg lateral views.

**Results:**

One hundred and twenty subjects were finally included, with 40 SCFE patients (36 males, 4 females) and 80 controls (72 males, 8 females). There were no significant differences in gender, age, weight, height, BMI, Hb, albumin (ALB), creatinine (Cr), free triiodothyronine (FT3), thyroid stimulating hormone (TSH), Ca, and P (*P*>0.05). Significant differences were found in 25(OH)D, ALP, free thyroxine (FT4), and Mg (*P*<0.05). The SCFE group had lower 25(OH)D and ALP levels but higher FT4 and Mg. Univariate analysis showed that 25(OH)D, FT4, and ALP were associated with SCFE, but multivariate analysis indicated only 25(OH)D had a significant correlation (*P*<0.05). 25(OH)D levels were not linked to SCFE severity (*P*>0.05).

**Conclusions:**

The results of this study indicate that a higher level of vitamin D is associated with a decreased risk of SCFE, suggesting potential benefits of vitamin D sufficiency. However, no correlation was observed between 25(OH)D levels and the severity of SCFE. Serum FT4 and ALP also seem to have some association with SCFE, but the clinical significance is unproven. Future multi-center studies in various regions are necessary to further validate the protective role of vitamin D against SCFE.

## 1 Introduction

SCFE mainly affects the hips of elder children and adolescents. It manifests as the relative displacement of the capital epiphysis of the femoral head in relation to the proximal femoral metaphysis. This displacement takes place when acute or chronical shear force works on a pathological physis.

The majority of SCFE occurred in children aging 10-14 years, especially those that are overweight or obese (1, 2). There is a slight male dominance. The incidence of SCFE varied slightly by region. In Sweden, the average annual incidence from 2007 to 2013 was 0.44 per 100000 girls and 0.57 per 100000 boys aging 9–15 years (1). In South Korea, SCFE showed an overall increasing trend in incidence, rising from 0.96 per 100000 in 2009 to 2.05 per 100000 in 2019 (3). Most SCFE patients presented with long-lasting dull pain around the hip and limping without specific traumatic history (4). Acute hip pain, inability to bear weight and limited range of motion may present when there is an acute aggravation of the slip. SCFE may lead to complications such as avascular necrosis of the femoral head, growth arrest and chondrolysis (4). Therefore, it is of great significance to explore the factors associated with the risk of SCFE, aiming to prevent its progression and improve the prognosis.

The etiology of SCFE includes biomechanical, histochemical and endocrinologic factors such as obesity and endocrine abnormality (5, 6). Obesity may promote skeletal maturation and increase the mechanical load on the physeal to trigger the slip. Additionally, it disrupts normal metabolism such as serum leptin and promote early puberty, which may contribute to the incidence of SCFE (6). Endocrine diseases, such as hyper-and hypothyroidism, hypogonadism, and hypopituitarism could also increase the risk of SCFE by directly or indirectly influencing hormone levels that are relevant to physis development (7). SCFE also demonstrated seasonal variation with incidence peaking in late summer or early autumn (8, 9). This seasonal change was more prominent in northern regions and less evident in area with minimal fluctuations in annual average temperature (8, 9). This variation was related to the latitude, skin color, sunlight exposure, seasonal variation of growth and intensity of growth plates and potentially the seasonal synthesis of VD (8, 9).

Vitamin D (VD) plays a crucial role in maintaining the homeostasis of the human body, particularly within the musculoskeletal system (10). The classical role of VD is to regulate calcium and phosphate metabolism and stimulate protein expression in the intestinal wall to enhance calcium absorption. VD is essential for optimizing bone health in childhood (11). Recent clinical studies have reported a high prevalence of VD deficiency in cases of fractures (12) and idiopathic scoliosis (13) among children and adolescents. Additionally, animal studies have demonstrated that VD plays a regulatory role in cartilage development, including the growth plate (14–16). In patients with SCFE, the growth plate was thicker with disrupted extracellular matrix and disarranged physis (17), which is somehow similar to that of vitamin D deficiency (16, 17). Therefore, it is reasonable to hypothesize that VD may play a role in the biological or pathological development of the proximal femoral growth plate. In recent years, increasing researches have explored the relationship between VD and SCFE (18–22). Most children with SCFE have insufficient or deficient levels of VD, suggesting that VD deficiency may be one of the risk factors for SCFE (18, 19, 21, 22). One study found that obese children with SCFE have a higher rate of VD deficiency compared to non-obese controls (22), while another study suggested that low VD level is uncorrelated to the development of SCFE in obese children (20). Therefore, the independent effect of VD on SCFE remains controversial.

Based on a retrospective case-control design, this study aimed at investigating the independent role of VD level in SCFE. Some other relevant factors that may be associated with the incidence and severity of SCFE were also included and analyzed.

## 2 Methods

This retrospective case-control study (1:2 pair-wise matching) was conducted in Shengjing hospital from March 2014 to October 2023, with the approval of the ethics committee (No.2024PS214K).

### 2.1 Subjects

Medical data of all SCFE patients from the Department of Pediatric Orthopedics were collected. The inclusion criteria were: (1) the patients are younger than 16 years old; (2) the patients were diagnosed SCFE when other musculoskeletal diseases had been ruled out; (3) the patients received biochemical tests including regular blood test, serum 25(OH)D level and bone-related nutrients measurement; (4) the patients received relevant hormones measurements.

Data of the control group was collected from the Department of Developmental Pediatrics. They were matched with the case group in terms of gender, age, BMI, and date of blood tests. All the subjects were free of any diagnosed diseases, but in order to match with the SCFE group, some participants were overweight or obese. All the subjects selected received the same tests as the SCFE patients.

The exclusion criteria were: (1) VD supplementation in six months before the blood test; (2) presence of other systems diseases that may affect physeal health or VD metabolism such as hypothyroidism and precocious puberty; (3) long-term use of medications that may affect physeal health or VD metabolism such as anticonvulsive drugs; (4) lack of complete medical record.

### 2.2 Demographic and laboratory data

General information of participants included gender, age, height and weight, BMI (BMI= mass(kg)/height(m)^2^) and date of blood test.

The sample for regular blood test was whole blood, while serum was used for all other tests. The test results were provided by the Department of Clinical Laboratory at Shengjing Hospital. Levels of Hb and serum ALB were included to reflect overall nutritional status. Serum Cr was included as an indicator of renal function. Levels of serum Ca, P, Mg and ALP were measured to evaluate bone metabolism. FT3, FT4 and TSH were measured to identify patients with thyroid dysfunction. Sex-related hormones, growth hormone and adrenal gland hormones were tested to assist in the diagnosis of abnormal gonadal and adrenal development.

The levels of serum 25(OH)D were measured by electrochemiluminescence immunoassay. Based on the results, VD status was classified into three levels: sufficiency (≥30 ng/mL), insufficiency (21-29 ng/mL), and deficiency (<20 ng/mL) (23).

### 2.3 Assessment of SCFE

**Figure 1:**
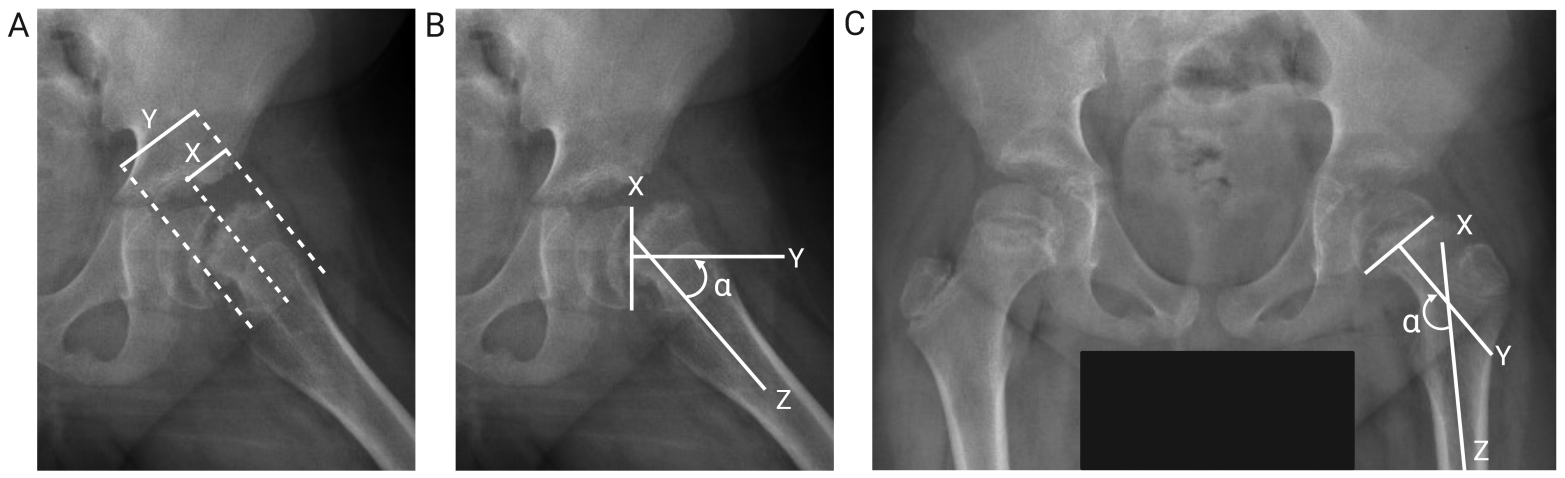
Diagram of measurement assessment criteria. **(A)** Epiphyseal displacement: the proportion of epiphyseal displacement (X) relative to the width of the femoral neck (Y) in the frog-leg lateral view. **(B)** HSA: the angle (α) between the mid-perpendicular line (Y) drawn through the line connecting the medial and lateral sides of the femoral epiphyseal plate (X) and the axis of the femoral shaft (Z) in the frog-leg lateral view. **(C)** EDA: the angle (α) between the mid-perpendicular line (Y) drawn through the line connecting the medial and lateral sides of the femoral epiphyseal plate (X) and the axis of the femoral shaft (Z) in the anteroposterior view.

The severity of SCFE was determined by measuring the slip angle and displacement in the anteroposterior pelvis and the frog-leg lateral views. Currently, the generally accepted assessments were proposed by Wilson and Southwick. Wilson et al. (24) described an assessment method based on the proportion of epiphyseal displacement on the femoral neck on the frog-leg lateral view (**Figure 1A**). Southwick et al. (25, 26) measured the epiphysis-diaphyseal angle (EDA) on the frog-leg lateral view (**Figure 1B**) and the proximal femoral head-shaft angle (HSA) on the anteroposterior view (**Figure 1C**). HSA can only be used in unilateral SCFE, as the severity of SCFE is defined according to the difference in HSA between the two sides.

A senior pediatric orthopedist (>20-year experience in pediatric orthopedics), a junior pediatric orthopedist (>10-year experience in pediatric orthopedics) and an orthopedic resident (<5-year experience in pediatric orthopedics) performed the measurements independently, and the mean of the three results was calculated to reduce observer bias. Discrepant results (where the proportion of epiphyseal displacement on the femoral neck differed by more than 5% or the EDA and HSA values differed by more than 5 degrees) were finally confirmed by another senior pediatric orthopedist.

Severity of SCFE were assessed using all the three methods, except for bilateral cases that cannot be assessed with HSA. The criteria are as follows:

1. Mild:

 • proportion of epiphyseal displacement<1/3

 • EDA<30°

 • Difference in bilateral HSAs < 30°

2. Moderate:

 • Proportion of epiphyseal displacement between 1/3 and 1/2

 • EDA between 30° and 50°

 • Difference in bilateral HSAs between 30° and 60°

3. Severe:

 • Proportion of epiphyseal displacement > 1/2

 • EDA > 50°

 • Difference in bilateral HSAs > 60°

### 2.4 Statistical analysis

Data were analyzed using SPSS software version 26.0 (SPSS Inc., Chicago, Illinois). All variables were tested for normal distribution. Normally distributed data were expressed as mean ± standard deviation, and comparisons between groups were performed using the *t* test. Data that did not follow a normal distribution were presented using the median (Q1, Q3), and comparisons between groups were performed using the non-parametric test. Data with inter-group differences were further evaluated for their potential association with SCFE using univariate and multivariate conditional logistic regression analyses. Finally, both linear (Pearson correlation) and nonlinear models were employed to assess the relationship between the levels of these factors and the severity of SCFE. *P*-values <0.05 were considered statistically significant.

## 3 Results

### 3.1 General data

Finally, this study enrolled a total of 40 patients (36 males, 4 females) with SCFE. The mean age of the patients was 10.6 ± 2.3 years, and their BMI was 26.5 ± 4.0 kg/m². Thirty-six cases of SCFE were unilateral (refer to **Table 1** for basic information), while four cases were bilateral (refer to **Table 2** for basic information). Accordantly, 80 healthy controls (72 males, 8 females) were matched. The mean age of the control group was 10.8 ± 1.9 years, with a mean BMI of 26.2 ± 3.6 kg/m².

**Table 1:** Basic information of unilateral SCFEs.

	Males (n=32)	Females (n=4)	Total (n=36)
25(OH)D			
**Sufficient**	1 (2.8%)	0 (0.0%)	1 (2.8%)
**Insufficient**	30 (83.3%)	3 (8.3%)	33 (91.6%)
**Deficient**	1 (2.8%)	1 (2.8%)	2 (5.6%)
Side			
**Left**	13 (36.1%)	2 (5.6%)	15 (41.7%)
**Right**	19 (52.7%)	2 (5.6%)	21 (58.3%)
Type			
**Stable**	27 (75.0%)	3 (8.3%)	30 (83.3%)
**Unstable**	5 (13.9%)	1 (2.8%)	6 (16.7%)
Severity of the slip displacement (proportion)			
**Mild**	15 (41.6%)	2 (5.6%)	17 (47.2%)
**Moderate**	8 (22.2%)	2 (5.6%)	10 (27.8%)
**Sever**	9 (25.0%)	0 (0.0%)	9 (25.0%)
Severity of the EDA			
**Mild**	1 (2.8%)	0 (0.0%)	1 (2.8%)
**Moderate**	16 (44.4%)	1 (2.8%)	17 (47.2%)
**Sever**	15 (41.7%)	3 (8.3%)	18 (50.0%)
Severity of the difference in bilateral HSAs			
**Mild**	25 (69.4%)	2 (5.6%)	27 (75.0%)
**Moderate**	7 (19.4%)	1 (2.8%)	8 (22.2%)
**Sever**	0 (0.0%)	1 (2.8%)	1 (2.8%)

Bolded values indicate statistical significance (P < 0.05).

**Table 2:** Basic information of bilateral SCFEs.

	Right condition (n=4)	Left condition (n=4)	Total (n=8)
Type			
**Stable**	3 (37.5%)	2 (25.0%)	5 (62.5%)
**Unstable**	1 (12.5%)	2 (25.0%)	3 (37.5%)
Severity of the slip displacement (proportion)			
**Mild**	4 (50.0%)	2 (25.0%)	6 (75.0%)
**Moderate**	0 (0.0%)	1 (12.5%)	1 (12.5%)
**Sever**	0 (0.0%)	1 (12.5%)	1 (12.5%)
Severity of the EDA			
**Mild**	2 (25.0%)	2 (25.0%)	4 (50.0%)
**Moderate**	1 (12.5%)	0 (0.0%)	1 (12.5%)
**Sever**	1 (12.5%)	2 (25.0%)	3 (37.5%)

All four cases of bilateral SCFE were male, with one VD deficiency and three VD insufficiency.Bolded values indicate statistical significance (P < 0.05).

### 3.2 Association between laboratory data and SCFE

Data of bodyweight, Hb, ALB, Cr, P, Mg, 25(OH)D, FT3, FT4 and TSH were normally distributed, whereas data of age, height, BMI, ALP, and Ca followed a non-normal distribution. After comparing the two groups, statistically significant differences were observed in the levels of 25(OH)D, ALP, Mg and FT4, as shown in **Table 3**.

**Table 3:** Comparison between the SCFE group and the control group.

	SCFE (n=40)	Control (n=80)	*P*
**Age (years old)**	11.000 (10.000, 12.000)	11.000 (10.000, 12.000)	0.257
**Height (cm)**	159.000 (147.000, 164.000)	153.000 (144.500, 160.000)	0.262
**Weight (kg)**	62.213 ± 14.803	60.269 ± 11.640	0.434
**BMI (kg/m^2^)**	26.0 00 (25.000, 27.400)	26.400 (24.910, 27.930)	0.641
**25(OH)D (ng/mL)**	19.027 ± 6.374	23.109 ± 5.489	**<0.001**
**Hb (g/L)**	136.00 ± 8.231	137.410 ± 8.500	0.423
**ALB (g/L)**	45.540 ± 3.438	45.763 ± 2.189	0.725
**ALP (U/L)**	200.000 (169.000, 258.000)	278.000 (238.000, 318.000)	**0.001**
**Ca (mmol/L)**	2.430 (2.340, 2.530)	2.370 (2.300, 2.430)	0.405
**Cr (μmol/L)**	41.773 ± 8.458	44.284 ± 8.621	0.182
**P (mmol/L)**	1.682 ± 0.140	1.647 ± 0.123	0.166
**Mg (mmol/L)**	0.920 ± 0.057	0.885 ± 0.101	**0.030**
**FT3 (pmol/L)**	5.420 ± 0.894	5.768 ± 0.701	0.074
**FT4 (pmol/L)**	14.697 ± 2.328	13.061 ± 1.297	**0.001**
**TSH (μIU/mol)**	2.251 ± 1.060	2.566 ± 1.110	0.226

Normally distributed data were expressed as mean ± standard deviation. Data that did not follow a normal distribution were presented using median (Q1, Q3).Bolded values indicate statistical significance (P < 0.05).

Next, the univariate conditional logistic regression analysis was performed on 25(OH)D, Mg, FT4 and ALP. 25(OH)D, FT4, and ALP were associated with the occurrence of SCFE (*P*<0.05), as presented in **Table 4**. While in the multivariate conditional logistic regression analysis, only 25(OH)D remained significantly associated (*P*<0.05), as shown in **Table 5**. The odds ratio (OR) for 25(OH)D was 0.779 (95% confidence interval, CI 0.610-0.995), indicating that a higher level of 25(OH)D were associated with a reduced risk of SCFE.

**Table 4:** Univariate conditional logistic regression analysis.

	B	SE	Z	OR	*P*	95%CI
**25 (OH) D**	-0.238	0.065	13.403	0.788	**<0.001**	0.694-0.895
**FT4**	0.503	0.207	5.912	1.653	**0.015**	1.102-2.497
**ALP**	-0.017	0.005	13.584	0.983	**<0.001**	0.974-0.992
**Mg**	5.623	3.071	3.353	276.593	0.067	0.673-113639.960

B, coefficients; SE, standard error; Z, Wald, Z=B/SE; OR, Odds Ratio.Bolded values indicate statistical significance (P < 0.05).

**Table 5:** Multivariate conditional logistic regression analysis.

	B	SE	Z	OR	*P*	95%CI
**25 (OH) D**	-0.250	0.125	4.002	0.779	**0.045**	0.610-0.995
**FT4**	0.188	0.344	0.296	1.206	0.586	0.614-2.369
**ALP**	-0.012	0.009	1.751	0.988	0.186	0.970-1.006

B, coefficients; SE, standard error; Z, Wald, Z, B/SE; OR, Odds Ratio.Bolded values indicate statistical significance (P < 0.05).

### 3.3 Association between 25(OH)D levels and the severity of SCFE

We further investigated the relationship between the level of 25(OH)D and the severity of SCFE. For bilateral SCFEs, the more severe side was used to represent its severity.

**Figure 2:**
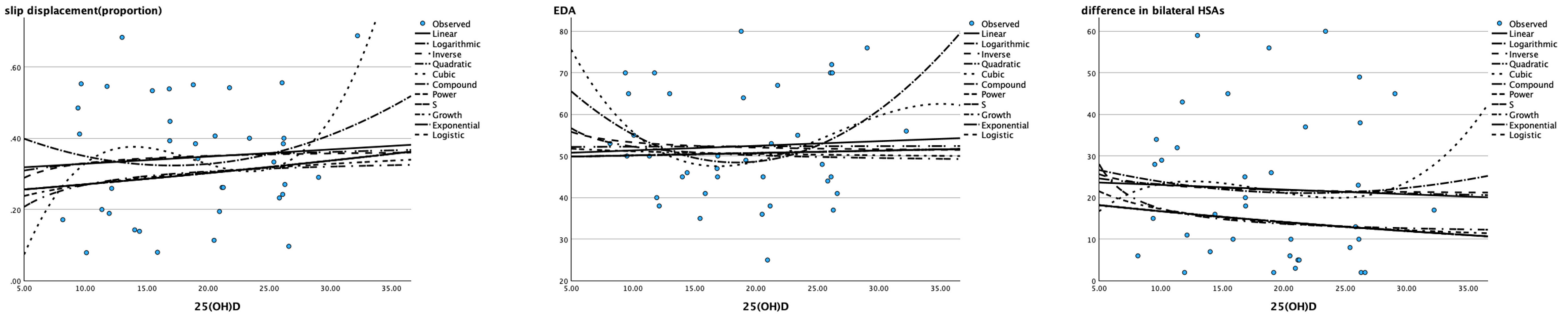
Scatter plot of 25(OH)D (X axis) and severity of SCFE (Y axis) The X-axis represents the 25 (OH) value of children with SCFE, and the Y-axis represents their severity (slip displacement described by proportion, EDA and the difference in bilateral HSAs). None of the models in the diagram could match their correlation.

Linear (Pearson correlation) and nonlinear models including the logarithmic, inverse, quadratic, cubic, compound, power, S curve, growth, exponential and logistic models were all not suitable for describing the relationship between the level of 25(OH)D and the severity of SCFE (all *P* > 0.05), as shown in **Table 6** and **Figure 2**.

**Table 6:** Correlation analysis between 25(OH)D level and SCFE severity.

Model		The Severity of SCFE		
		Slip Displacement	EDA	Difference in Bilateral HSAs
P	Linear/Pearson	0.736	0.688	0.873
	Logarithmic	0.815	0.933	0.890
	Inverse	0.862	0.816	0.952
	Quadratic	0.617	0.193	0.977
	Cubic	0.374	0.256	0.900
	Compound	0.571	0.720	0.690
	Power	0.629	0.980	0.680
	S curve	0.681	0.758	0.723
	Growth	0.571	0.720	0.690
	Exponential	0.571	0.720	0.690
	Logistic	0.571	0.720	0.690

## 4 Discussion

SCFE is a multi-factorial disease that is associated with personal characteristics, behaviors and environment. This study was designed to reduce the influence of confounding factors by matching variables including gender, age, weight, height, BMI, and the date of blood tests. The influence of endocrine abnormalities was also eliminated. Our study showed significant differences in 25(OH)D, ALP, FT4, and Mg (*P*<0.05) between SCFE patients and the control group. Further univariate conditional logistic regression revealed that 25(OH)D, ALP, and FT4 were associated with the risk of SCFE (*P*<0.05), while Mg was not (*P*>0.05). Multivariate conditional logistic regression analysis demonstrated that only 25(OH)D was significant (*P*<0.05). Univariate conditional logistic regression initially identified variables associated with SCFE risk, while multivariate conditional logistic regression, controlling for other factors, indicated that the optimal level of VD might be a protective factor against SCFE with the OR of 0.779. As the level of 25(OH)D increased, the risk of SCFE decreased (*P*<0.05).

This conclusion was consistent with the findings of several other studies (18, 19, 22). Madhuri et al. (18) first suggested that SCFE was not only related to high BMI but also to VD deficiency. Judd et al. (19) observed that most children diagnosed with SCFE were VD deficient. They also noted that in children with VD deficiency who underwent *in situ* fixation with cannulated screws, there was a varying degree of delay in the time of the growth plate closure. Natalie et al. (22) found that VD deficiency was a risk factor for not only the incidence of SCFE but also post-slip osteonecrosis.

Our study found that serum 25(OH)D level was not correlated with the severity of SCFE (*P*>0.05), which was consistent with the findings of Elbeshry et al. (21), but contrary to those of Judd et al (19). Elbeshry et al. (21) found no significant relationship between VD levels and the lateral Southwick angle in the frog-leg view in 39 children with SCFE. In contrast, Judd et al. (19) conducted a retrospective cohort study on 27 children with SCFE and found that low VD levels were associated with greater slip severity reflected by the posterior sloping angle and prolonged time to physeal fusion. Both these two studies used a single method to judge the severity of SCFE. While in this study, we used three different methods to assess the severity of SCFE, providing more solid evidence that there was no significant relationship between the VD level and the severity of SCFE.

Seasonal variation in VD levels among children and adolescents was also observed in this study. In both the SCFE group and the control group, higher VD levels were observed in the summer (SCFE group average: 22.53 ng/ml, control group average: 27.34 ng/ml) and autumn (SCFE: 21.64 ng/ml, control: 22.67 ng/ml) compared to spring (SCFE: 15.40 ng/ml, control: 21.32 ng/ml) and winter (SCFE: 15.60 ng/ml, control: 22.05 ng/ml). Similar trend had also been observed in pediatric patients with upper limb fractures (12). However, the seasonal variation in the incidence or severity of SCFE was not observed in this study, possibly due to the limited size of the sample.

Although there is a lack of mechanical research on how VD directly affects SCFE, some animal studies have provided indirect evidence about their relationship. The pathology of SCFE is characterized by irregular cluster arrangement of chondrocytes in the thickened growth plate with an altered ratio between the thickness of the resting zone and the proliferating, maturation, hypertrophic and degenerating zones (17, 27). A comparison of the proximal tibial growth plate in VD-deficient mice and the growth plate of SCFE showed similar structural disorganization, including changes in chondrocyte arrangement and irregular widening of the proliferative and hypertrophic zones (16, 17). In porcine animal experiments, it was found that the VD-deficient group showed lower tolerance to shear stress and strain compared to the VD-normal group (14). Other murine experiments confirmed that VD supplementation had a favorable impact on the development of articular cartilage thickness, joint lubrication, and extracellular matrix fiber deposition (15). These implies that VD might influence the incidence of SCFE by working on the development and function of the physis. However, possibly due to the difficulties in establishing an animal model of SCFE, the confirmation of this hypothesis still requires further efforts.

Ca, P, and Mg are all important ions that are involved bone growth and metabolism, and their serum levels generally fluctuate within a normal reference range (28). In this study, only one child with SCFE and three children in the control group had slightly lower serum Ca levels, and two children with SCFE had slightly elevated serum P levels. Those slight variations are believed to be natural among the population. This suggested that the metabolism of Ca and P are not disturbed in SCFE cases. Although there was a significant difference in serum Mg levels between the two groups (*P*<0.05), subsequent univariate logistic regression analysis showed no association between Mg levels and the risk of SCFE (*P*>0.05). Therefore, it was believed that serum levels of Ca, P, and Mg had no significant impact on the risk of SCFE.

In addition to 25(OH)D, FT4 and ALP were found to be associated with the risk of SCFE. The univariate logistic regress analysis showed that the OR of FT4 was 1.653 (*P*=0.015), and the OR of ALP was 0.983 (*P*<0.001), which indicated that FT4 level is positively correlated with the risk of SCFE, while the ALP level is negatively correlated. The clinical significance of this result, however, remains unclear. Imbalance of thyroid hormones, sex hormones, growth hormones, and adrenal hormones may be associated with the risk and development of SCFE (29), among which hypothyroidism is the most common (30). The thyroid hormones may be regulated by gender, age, BMI and puberty (31, 32). Hypothyroidism generally presents with low levels of thyroxine (T4) and triiodothyronine (T3), and a high level of TSH. T4 includes both the bound and the free forms (FT4). Although the concentration of FT4 in circulation is low, it is of high diagnostic value and is widely used as an indicator of T4 level in clinical practice. The average FT4 level in SCFE patients (14.697 ± 2.328 pmol/L) was about 1.6 pmol/L higher than in the control group (13.061 ± 1.297 pmol/L). But according to an survey of 1279 Chinese healthy children (33), only one 11-year-old male SCFE patient in this study had an FT4 level (9.97 pmol/L) below the normal range, while the rest were all within the normal range (12.65-23.16 pmol/L). Elevated FT4 levels with normal TSH are rare in children and are typically associated with TSH suppression and hyperthyroidism (34), but none of the SCFE cases in this study met the clinical criteria of hyperthyroidism. The elevation in FT4 levels might be related to SCFE, but its clinical significance is still unclear. A larger sample is needed to confirm the clinical significance of this phenomenon. Meanwhile, current evidence is still insufficient to clarify its exact mechanism.

We also observed the decreased serum ALP level in SCFE patients compared to controls, and further analysis indicated a negative relation between serum ALP level and SCFE. Most of the serum ALP in children comes from the bone, and ALP level in children during their active growth periods can be 1.5-2.5 times that of adults (35). Mice with non-specific ALP knockout exhibited insufficient dephosphorylation of matrix phosphoproteins, delayed or blocked bone mineralization, disrupted epiphyseal development, and reduced number of hypertrophic chondrocytes (36). This was consistent with the microscopic changes in SCFE observed by Tresoldi (17). Decreased ALP level may imply decreased activity of the physeal chondrocytes, or disrupted physeal development that predispose the subject to capital epiphysis displacement. However, there are no basic studies directly investigating the relationship between ALP and SCFE. Furthermore, many factors can affect serum ALP levels, such as age, gender, overall developmental status and hepatobiliary and hematological diseases (37). Further statistical analysis that can eliminate those confounding factors may be necessary to demonstrate the role of ALP in SCFE.

This study has several limitations (1): Although this study has a relatively large sample size among studies on SCFE, the number of cases is limited and all the cases come from one research center (2); Despite being matching by age, gender, weight, and height, and excluding six SCFE patients diagnosed with hypothyroidism, it was still challenging to control for all variables such as the degree of gonadal development. Some matching was based on BMI, resulting in higher average weight and BMI in the control group compared to the normal population. Further prospective, multi-center studies are needed to confirm the protective role of VD in SCFE. Such studies would include more SCFE cases from different latitude and more healthy controls to match, enhancing the reliability of the conclusion and ensuring their applicability across diverse populations and clinical settings.

## 5 Conclusion

In conclusion, this study found that vitamin D deficiency is more severe in patients with SCFE, indicating that lower serum 25(OH)D level may be correlated with increased occurrence of SCFE. It supports a potential protective role of vitamin D against SCFE, though no association between the severity of vitamin D deficiency and the severity of SCFE was found. Serum FT4 and ALP also seems to be correlated with the incidence of SCFE, but the clinical significance of this finding remains to be investigated. Future multi-center studies including centers of different latitude are necessary to further validate the role of vitamin D in SCFE.

## Data Availability

The raw data supporting the conclusions of this article will be made available by the authors, without undue reservation.
